# A simple risk index predicts endovascular treatment outcomes in acute ischemic stroke: prognostic value of SPAN-100

**DOI:** 10.3389/fneur.2025.1578997

**Published:** 2025-07-07

**Authors:** Noémie Ligot, Marie Dagonnier, Boris Lubicz, Nicolas Brassart, Gilles Naeije

**Affiliations:** ^1^Department of Neurology, Hôpital Erasme, Université Libre de Bruxelles, Brussels, Belgium; ^2^Department of Neurology, CHU HELORA—Kennedy, Mons, Belgium; ^3^Department of Interventional Neuroradiology, Hôpital Erasme, Université Libre de Bruxelles, Brussels, Belgium; ^4^Department of Interventional Vascular Radiology, CHU HELORA—Kennedy, Mons, Belgium

**Keywords:** acute ischemic stroke, mechanical thrombectomy, SPAN-100 index, prognosis, modified Rankin scale, older individuals

## Abstract

**Background:**

Mechanical thrombectomy (MT) is a proven intervention for patients with acute ischemic stroke (AIS) due to a large vessel occlusion (LVO). However, outcomes after MT remain variable, particularly in high-risk groups. The SPAN-100 index, a simple bedside tool combining age and baseline NIHSS scores, has been associated with poor outcomes in AIS patients treated with intravenous thrombolysis. This study evaluates the prognostic value of the SPAN-100 index and its weighted variant (wSPAN) in predicting outcomes following MT.

**Methods:**

We conducted a retrospective cohort study of patients with AIS who were treated with MT between 2015 and 2024 at two Belgian university hospitals. SPAN and wSPAN scores were calculated at admission, and patients were stratified by SPAN ≥100 vs. <100. The primary outcome was functional status at 90 days, as assessed using the modified Rankin Scale (mRS). Secondary outcomes included mortality (mRS = 6) and favorable outcomes (mRS ≤ 3). Discriminative ability was assessed using receiver operating characteristic (ROC) analysis (AUC, 95% CI), and model performance was evaluated using AIC and BIC. The positive predictive value (PPV) and negative predictive value (NPV) were calculated for SPAN ≥100, and outcome proportions were compared between SPAN-defined groups using Fisher’s exact test.

**Results:**

A total of 530 patients were included, of whom 116 had SPAN scores of ≥100. These patients experienced significantly worse outcomes, with higher mortality (60.0% vs. 17.6%) and lower rates of mRS ≤ 3 (19.2% vs. 71.6%, both *p*-values < 0.001). However, among survivors with SPAN scores of ≥100, nearly half of them achieved recovery (mRS ≤ 3). ROC analysis showed good discrimination for both SPAN and wSPAN: AUCs were 0.77 and 0.78 for mRS ≤ 3 and 0.80 and 0.81 for mortality. wSPAN showed a slightly better model fit (lower AIC/BIC). The SPAN ≥100 threshold had a high PPV for poor outcomes and a high NPV for survival.

**Conclusion:**

SPAN-100 and wSPAN are pragmatic and reliable prognostic tools for AIS patients undergoing MT. While a SPAN score ≥100 identifies a high-risk group with poorer overall outcomes, it should not be used as an exclusion criterion. Many patients with a SPAN score ≥100 achieved functional recovery, supporting MT as a justified intervention even for older, more severely affected individuals. These findings highlight the importance of combining risk stratification with clinical judgment rather than relying on rigid thresholds.

## 1 Introduction

Mechanical thrombectomy (MT) has revolutionized the treatment of acute ischemic stroke (AIS) caused by large vessel occlusion (LVO), offering substantial improvements in functional outcomes when performed promptly. Despite the rapid evolution of mechanical thrombectomy (MT) as the standard of care for acute ischemic stroke due to large vessel occlusion, outcome prediction remains challenging. Numerous multivariable models have been proposed, incorporating clinical variables (1, 2), advanced neuroimaging (core infarct volume, ASPECT, collateral status, MRI-based penumbra) (3–6), or procedural metrics (e.g., time to recanalization) (5, 7). However, even when combining these features, the added prognostic value remains limited, and model performance is often suboptimal in external validation (1, 2). More importantly, such models are frequently complex, require unavailable or delayed inputs, and lack bedside applicability. This complexity underscores the need for reliable, practical prognostic tools to assist clinicians in making informed treatment decisions and to guide discussions with patients and their families about expected outcomes in these high-stakes scenarios (8, 9).

The Stroke Prognostication using the age and NIH Stroke scale (SPAN-100) index is a simple and pragmatic tool that sums age and baseline National Institutes of Health Stroke Scale (NIHSS) score to estimate stroke outcomes. Patients with SPAN-100 scores of 100 or greater (SPAN≥100) are typically older with more severe neurological deficits, characteristics that have been consistently associated with poorer prognoses. Studies in patients treated with intravenous thrombolysis (IVT) have demonstrated that SPAN ≥100 status predicts higher rates of mortality and severe disability at 90 days (10, 11). However, the applicability of the SPAN-100 index in patients undergoing MT remains seldom underexplored (12). This knowledge gap is particularly important (13) given that MT is a highly effective intervention for LVO, even in some high-risk populations (14) and when subgroups are weighted for age/NIHSS, despite the combined adverse effect of age and NIHSS on MT outcomes (13).

Understanding the role of SPAN-100 in MT could refine prognostication and enhance decision-making by helping clinicians balance the risks and benefits of treatment for patients with complex profiles. Furthermore, prognostic tools such as SPAN-100 provide a valuable framework for counseling patients and their families about expected functional outcomes, fostering shared decision-making and realistic goal setting in the post-stroke recovery process. By identifying high-risk patients who might still benefit from MT, the SPAN-100 index could optimize resource allocation and improve individualized care strategies in AIS management.

This study aims to evaluate the SPAN-100 index, based solely on age and NIHSS, and its weighted variant (wSPAN, which consists of the sum of age added to three times the NIHSS) (13) as practical bedside tools for prognostication in a real-world multicenter thrombectomy cohort. The SPAN framework is intentionally minimalistic but anchored on the two most influential and universally available predictors of stroke outcome. We hypothesized that SPAN and wSPAN would demonstrate discriminative performance comparable to more elaborate models, thus offering a pragmatic solution for rapid risk stratification.

## 2 Methods

### 2.1 Study design and population

This retrospective cohort study included consecutive patients with acute ischemic stroke (AIS) due to anterior circulation large vessel occlusion (LVO) who were treated with mechanical thrombectomy (MT) at HUB Hôpital Erasme, Université Libre de Bruxelles, and CHU HELORA—Kennedy, Mons, between January 2015 and January 2024. Patients were included if they met the following criteria: (i) age ≥18 years; (ii) presentation with anterior LVO, defined as occlusion of the internal carotid artery (ICA), middle cerebral artery (M1 or M2 segments), or anterior cerebral artery (A1 or A2 segments); and (iii) availability of complete pre- and post-thrombectomy clinical data. Patients with posterior circulation strokes or incomplete clinical data were excluded (15).

### 2.2 Prognostic assessment using SPAN-100

The SPAN-100 index was calculated for all patients by summing their age in years and their pre-treatment NIHSS score. Patients were categorized as SPAN≥100 if the combined score was ≥100 and as SPAN<100 if the score was <100 (10). The weighted SPAN was computed by adding the patient’s age to three times the NIHSS value (13).

### 2.3 Outcome measures

The primary outcome was functional status at 90 days, as assessed using the modified Rankin Scale (mRS). Poor outcomes were defined as mRS scores of 4–6, indicating severe disability or death, while favorable outcomes were defined as mRS scores ≤3, reflecting independence or moderate disability. Mortality (mRS = 6) was analyzed as a secondary outcome. Outcomes were evaluated during routine clinical follow-up or through structured telephone interviews with patients or their caregivers.

### 2.4 Statistical analysis

Descriptive statistics were used to summarize patient characteristics, stratified by SPAN-100 status. Statistical comparisons of the proportions of modified Rankin Scale (mRS) scores between the SPAN-100 groups were performed using the chi-squared test for each score. This method assesses whether the observed frequencies of categorical data differ significantly between groups. Since the analysis included seven mRS scores (0 through 6), a Bonferroni correction was applied to account for multiple comparisons (*n* = 7). The corrected significance threshold was calculated as *p* = 0.05/7 (0.0071).

To evaluate the diagnostic performance of the SPAN ≥ 100 threshold, we computed sensitivity, specificity, positive predictive value (PPV), and negative predictive value (NPV) for three clinical outcomes: favorable outcome defined as mRS ≤ 2 and ≤3, and poor outcome defined as mRS = 6. The same metrics were also calculated for the complementary group (SPAN < 100) to enable direct comparison.

To assess prognostic accuracy, we conducted receiver operating characteristic (ROC) curve analyses for SPAN and wSPAN scores, with outcomes defined as favorable (mRS ≤ 2 and ≤3) and mortality (mRS = 6). SPAN and wSPAN scores were inverted for the prediction of favorable outcomes to reflect their intended use as risk indices, where higher values indicate a worse prognosis. We calculated the area under the ROC curve (AUC) and the corresponding 95% confidence intervals using the DeLong method (16). To further evaluate and compare model performance, we computed the Akaike Information Criterion (AIC) and the Bayesian Information Criterion (BIC) from logistic regression models predicting each outcome. These information criteria quantify model quality by balancing goodness-of-fit with model complexity: lower values indicate a more parsimonious and better-fitting model. While the AIC applies a modest penalty for additional parameters, the BIC imposes a stricter penalty that increases with sample size. These criteria enable an objective comparison of non-nested models such as SPAN and wSPAN, which differ in the relative weighting of NIHSS versus age. A difference of 2–6 points between models is considered moderate evidence in favor of the lower-scoring model, while a difference greater than 10 points is considered strong evidence.

To quantify the relative contributions of age and NIHSS, we used logistic regression models with both variables as predictors and extracted the standardized effect coefficients. The NIHSS-to-age ratio was used to justify the weighting scheme applied in wSPAN (13).

### 2.5 Ethics

The study was conducted in accordance with the Declaration of Helsinki and was approved by the Ethics Committee of CUB Hôpital Erasme, Université Libre de Bruxelles, Brussels, Belgium. The retrospective nature of the study and the use of de-identified data obviated the need for informed consent under national legislation and institutional guidelines.

## 3 Results

### 3.1 Cohort

Of the 604 individuals who met the inclusion criteria, 60 were excluded due to missing mRS status at 3 months. The final cohort consisted of 530 individuals (241 men), with a mean age of 71 ± 15 years and a mean admission NIHSS score of 15 ± 7.

### 3.2 Outcome distribution by SPAN-100 status

**Figure 1:**
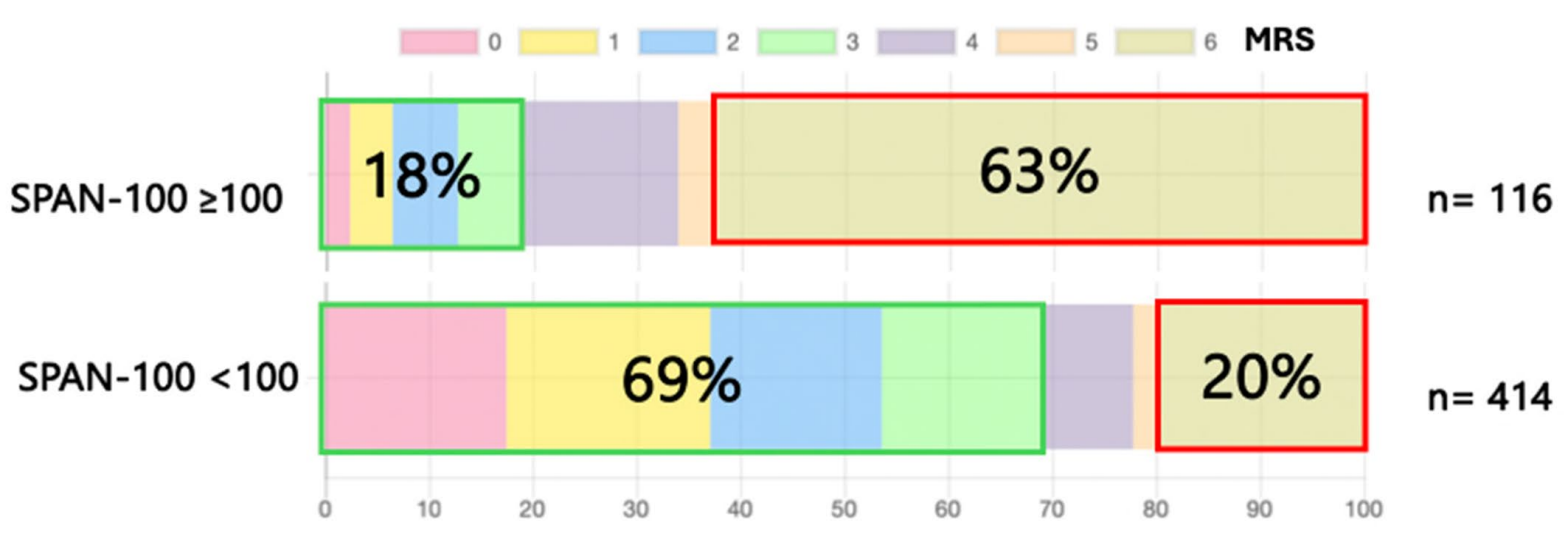
Three-month functional outcomes among patients with acute ischemic stroke due to anterior circulation large vessel occlusion. Outcomes are based on the modified Rankin Scale (from 0 [no symptoms] to 6 [death]) and stratified by the stroke prognostication using age and the NIH stroke scale index, created by combining age in years plus a National Institutes of Health (NIH) stroke scale score of 100 or higher (and referred to as the SPAN-100 index).

The analysis revealed stark differences in 90-day outcomes between the SPAN≥100 and SPAN<100 groups. Among SPAN≥100 patients, 63% experienced mortality (mRS = 6), highlighting the extremely high mortality risk in this group. However, of the SPAN≥100 patients who survived, 18% achieved favorable outcomes (mRS ≤ 3), demonstrating the potential for meaningful recovery in some high-risk patients. This finding highlights a distinct “all-or-nothing” pattern of outcomes for SPAN≥100 patients (Figure 1).

Conversely, in the SPAN<100 group, 69% of patients achieved favorable outcomes (mRS ≤ 3), with only 20% experiencing mortality (mRS 6). These findings illustrate a significantly better prognosis for SPAN<100 patients compared to their SPAN≥100 counterparts (Table 1). At the SPAN ≥ 100 threshold, the index showed high specificity for poor outcomes (specificity = 0.870) and a positive predictive value (PPV) of 0.600 for mortality (mRS = 6), indicating a strong association with death. The sensitivity for mortality was 0.430, and the negative predictive value (NPV) was 0.824. For favorable outcomes, SPAN ≥ 100 had low sensitivity (0.064 for an mRS ≤ 2; 0.076 for an mRS ≤ 3) and modest PPVs (0.125 and 0.192, respectively) but relatively high specificity (0.952 for an mRS ≤ 2; 0.929 for an mRS ≤ 3) and moderate NPVs (0.801 and 0.732, respectively). In contrast, SPAN < 100 demonstrated high sensitivity for identifying favorable outcomes (0.936 for an mRS ≤ 2; 0.924 for an mRS ≤ 3), with NPVs of 0.875 and 0.808, respectively. However, its specificity for poor outcomes was lower (0.570 for an mRS = 6), and its PPV for identifying favorable outcomes was also modest (0.472 for an mRS ≤ 2; 0.587 for an mRS ≤ 3). These findings confirm that SPAN ≥ 100 is more specific than SPAN < 100 for predicting poor outcomes, whereas SPAN < 100 possesses higher sensitivity than SPAN ≥ 100 for identifying patients with potential for recovery.

**Table 1:** Difference of MRS proportions between SPAN≥100 and SPAN<100 groups.

mRS score	Chi-squared	*p*-value
0	11.1	0.001
1	11.81	0.001
2	4.14	0.042
3	3.28	0.07
4	1.3	0.255
5	0.05	0.816
6	36.11	< 0.001

### 3.3 ROC analysis

ROC analysis showed that both SPAN and wSPAN exhibited strong discriminative ability. The AUC for predicting a favorable outcome (mRS ≤ 3) was 0.77 for SPAN and 0.78 for wSPAN. For mortality (mRS = 6), the AUC was 0.80 and 0.81, respectively. Logistic regression analyses confirmed that the NIHSS contributed more than twice the predictive weight of age (NIHSS/age coefficient ratio: 2.39 for an mRS ≤ 2, 2.29 for an mRS ≤ 3, and 2.10 for mortality), supporting the use of a weighted index. Model fit indicators (AIC, BIC) consistently favored wSPAN (Table 2).

**Table 2:** Summarizes models performances metrics.

Outcome	Predictor	AUC	95% CI	AIC	BIC
mRS ≤ 2	SPAN	0.757	(0.715–0.8)	607.4	615.8
mRS ≤ 2	wSPAN	0.765	(0.723–0.807)	589.5	598.0
mRS ≤ 3	SPAN	0.778	(0.738–0.817)	568.7	577.2
mRS ≤ 3	wSPAN	0.787	(0.748–0.825)	557.8	566.3
mRS = 6	SPAN	0.769	(0.719–0.818)	508.0	516.5
mRS = 6	wSPAN	0.776	(0.727–0.825)	501.0	509.5

mRS, modified Rankin Scale; AUC, area under curve; CI, confidence interval; AIC, Akaike Information Criterion; BIC, Bayesian Information Criterion.

**Figure 2:**
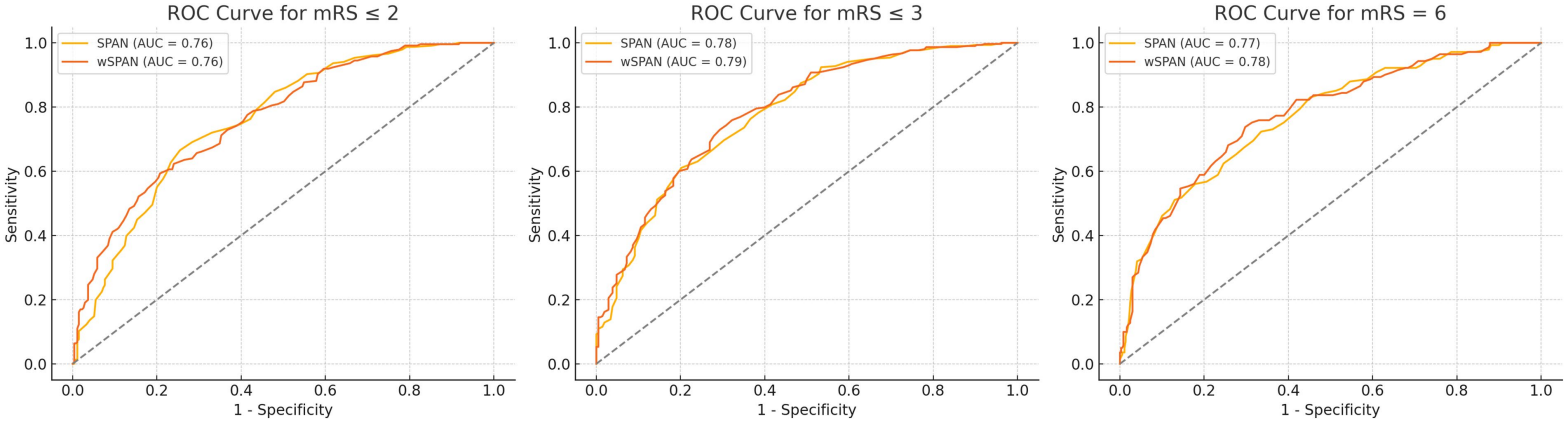
ROC curves of the SPAN and wSPAN for the different outcome measures. AUC, area under the curve.

Figure 2 illustrates the AUC for predicting different outcomes according to SPAN or wSPAN.

## 4 Discussion

This study evaluated the prognostic utility of the SPAN-100 index and its weighted variant (wSPAN) in patients undergoing mechanical thrombectomy (MT) for acute ischemic stroke (AIS) due to large vessel occlusion (LVO). Our findings confirm that a SPAN≥100 status is strongly associated with poor outcomes, including markedly higher rates of mortality and lower chances of functional independence. Specifically, patients with SPAN≥100 had a six-fold higher mortality rate (60.0% vs. 17.6%) and a substantially reduced likelihood of achieving an mRS ≤ 3 (19.2% vs. 71.6%, *p* < 0.001) compared to those with SPAN<100. SPAN ≥ 100 was highly specific for identifying poor outcomes, while SPAN < 100 was strongly associated with favorable outcomes, as reflected by its high negative predictive value.

While these results underscore the high mortality risk for SPAN≥100 patients, it is critical to emphasize the significant proportion of survivors who achieved relatively favorable functional outcomes. Among SPAN≥100 patients who survived, approximately 50% achieved an mRS of ≤3, indicating moderate to no disability and a potential for meaningful recovery. This striking “all-or-nothing” scenario—where SPAN≥100 patients either face high rates of mortality or achieve a reasonable degree of functional independence—should prompt clinicians to carefully consider the potential benefits of MT rather than disqualifying SPAN≥100 patients based solely on their risk profile. These findings reinforce that MT remains a lifesaving and function-restoring intervention, even in high-risk populations (8, 13).

The ability of SPAN≥100 patients to achieve functional recovery highlights the importance of patient selection and the nuanced application of prognostic tools such as the SPAN-100 index. While SPAN-100 is a useful marker for identifying patients at increased risk of poor outcomes, its application should not discourage clinicians from offering MT, especially when other favorable factors, such as early treatment initiation, high ASPECTS, or good collateral circulation, are present (14, 17). Instead, SPAN-100 should guide individualized discussions with patients and their families about the potential for both risks and benefits, particularly in the context of this “all-or-nothing” dynamic.

Our findings align with a growing body of evidence questioning the added value of complex prognostic models in MT and reinforce the practical strengths of SPAN and wSPAN. Kremers et al. (1) systematically reviewed 19 pre-MT prediction models, which included between 2 and 11 variables combining clinical, biological, comorbidity, and radiological characteristics. They then validated these models using data from the MR-CLEAN registry (*n* = 3,156) and found that all models performed within similar ranges of AUC. Interestingly, all the selected models included age and NIHSS as core predictors. The two best-performing models were the THRIVE-c, which included, besides age and NIHSS, only three comorbidities (hypertension, atrial fibrillation, and diabetes mellitus), achieving an AUC of 0.74, and the MR-PREDICTS model, which added imaging features (ASPECT, collaterals, and occlusion location) to baseline stroke characteristics and comorbidities, yielding a slightly better AUC of 0.8 (1). This large study suggested that comorbidities, imaging, and complex baseline characteristics contributed little beyond age and baseline NIHSS. Supporting these findings, Ospel et al. (2), using data from 1,105 patients of trial registry, showed that even the best-performing models—which incorporated post-treatment variables—achieved AUCs of 0.82–0.88. Their baseline-only pragmatic model, including age, NIHSS, onset-to-CT-time, ASPECTS, and occlusion site, performed similarly to their comprehensive baseline model that added patients’ comorbidities and treatment variables; both achieved AUCs of 0.74–0.78 for predicting good and poor outcomes. In all models, age and NIHSS were the most influential features, as shown by Shapley value analysis, with other factors contributing marginally (2).

Our models, based solely on age and NIHSS, achieved AUCs between 0.76 and 0.81, matching or exceeding those of many published models while maintaining complete bedside applicability (1, 2, 13). Unlike models requiring imaging (e.g., core volume and collaterals), perfusion mismatch, or post-treatment angiographic data, SPAN and wSPAN require no additional resources and can be implemented within minutes of clinical evaluation.

The findings of this study, albeit limited by its monocentric nature, are likely to be generalizable to other populations of LVO treated with mechanical thrombectomy (MT) within 6 h of symptom onset. Indeed, the cohort aligns closely with the characteristics of the pooled populations included in the meta-analysis of the five major trials that validated MT: MR CLEAN, ESCAPE, REVASCAT, SWIFT PRIME, and EXTEND IA (18). Specifically, patients in our cohort had a comparable median age, admission NIHSS, and rate of ICA and M1 occlusions, which are key determinants of stroke severity and thrombectomy outcomes (5, 18). The proportion of patients achieving favorable outcomes (mRS ≤ 3: 67%) was comparable to that reported in the validation trials (63%) (18).

These similarities in baseline characteristics and outcomes support the generalizability of our findings to broader thrombectomy populations. Moreover, the consistent association between SPAN≥100 status and poorer functional outcomes across studies, observed both in thrombolysis-focused research (10, 11) and in this thrombectomy-specific cohort, further validates the utility of the SPAN-100 index as a reliable prognostic tool. Importantly, while SPAN≥100 patients demonstrated higher mortality rates, our findings highlight that nearly 18% of SPAN≥100 patients who survived achieved functional independence (mRS ≤ 3), underscoring the importance of not excluding this high-risk group from thrombectomy based solely on their risk profile. These findings reinforce the relevance of SPAN-100 in clinical decision-making, offering a practical and generalizable tool for risk stratification in acute ischemic stroke.

From a clinical perspective, the SPAN-100 index provides a valuable framework for risk stratification and shared decision-making. For SPAN≥100 patients, discussions should incorporate the high likelihood of severe disability or mortality but also emphasize the realistic potential for meaningful recovery in survivors. This balanced approach can help clinicians counsel patients and their families effectively, ensuring that decisions reflect both the risks and the opportunities afforded by MT. Importantly, these findings should encourage clinicians to offer MT to SPAN≥100 patients rather than withholding treatment based on perceived futility. The potential for functional recovery in survivors supports a proactive approach, emphasizing individualized patient care and the use of MT as a life-saving therapy in this high-risk group.

Despite its strengths, our study has limitations. The retrospective design and reliance on registry data may introduce selection bias, and the generalizability of our findings to populations with different baseline characteristics requires further investigation. Additionally, models limited to baseline data will inherently have restricted predictive precision, and adding imaging, comorbidity, and post-treatment variables could improve discrimination, but at the cost of real-time usability. The SPAN-100 index, despite its simplicity, leverages the two most influential variables: age and NIHSS, which had the highest Shapley values in Ospel et al. (13) and are validated across all MT prediction models (1). On the other hand, the SPAN-100 remains clinically interpretable, reproducible, and implementable at the bedside or in pre-hospital triage. Therefore, we believe the present study contributes meaningfully by validating the practical prognostic value of SPAN in a multicenter thrombectomy cohort, but it does not replace more complex models that are developed to understand the full range of patients’ outcome variability.

## 5 Conclusion

In this multicenter cohort of patients treated with mechanical thrombectomy for large vessel occlusion stroke, both the SPAN-100 and its weighted variant (wSPAN) demonstrated strong prognostic value. A SPAN ≥100 status was associated with significantly higher mortality and lower rates of functional independence; however, nearly half of the survivors in this group achieved an mRS ≤ 3, underscoring that high risk does not equate to futility.

SPAN and wSPAN achieved AUCs comparable to or exceeding those of more complex models, while offering immediate bedside applicability without reliance on advanced imaging or post-treatment variables. These findings support the integration of SPAN-100 into clinical workflows as a simple, interpretable tool to guide risk communication and treatment decisions, ensuring that even high-risk patients are considered for thrombectomy when appropriate.

## Data Availability

The raw data supporting the conclusions of this article will be made available by the authors, without undue reservation.

## References

[1] Kremers F Venema E Duvekot M Yo L Bokkers R Lycklama À Nijeholt G. Outcome prediction models for endovascular treatment of ischemic stroke: systematic review and external validation. Stroke. (2022) 53:825–36. doi: 10.1161/STROKEAHA.120.033445, PMID: 34732070 PMC8884132

[2] Ospel JM Ganesh A Kappelhof M McDonough R Menon BK Almekhlafi M. Evaluating outcome prediction models in endovascular stroke treatment using baseline, treatment, and posttreatment variables. Stroke Vasc Interv Neurol. (2021) 1:1. doi: 10.1161/SVIN.121.000167 PMC12512352 41584466

[3] Elands S Casimir P Bonnet T Mine B Lubicz B Sjøgård M. Early venous filling following Thrombectomy: association with hemorrhagic transformation and functional outcome. Front Neurol. (2021) 12:649079. doi: 10.3389/fneur.2021.649079, PMID: 33776899 PMC7987949

[4] Favilla CG Regenhardt RW Denny B Shakibajahromi B Patel AB Mullen MT. Validation of a novel magnetic resonance imaging biomarker of infarct severity to predict functional outcome after endovascular Thrombectomy. Stroke. (2025) 56:926–36. doi: 10.1161/STROKEAHA.124.050508, PMID: 39882618

[5] Ligot N Elands S Damien C Jodaitis L Sadeghi Meibodi N Mine B. Stroke core volume weighs more than recanalization time for predicting outcome in large vessel occlusion recanalized within 6 h of symptoms onset. Front Neurol. (2022) 13. doi: 10.3389/fneur.2022.838192, PMID: 35265032 PMC8898898

[6] Ospel J Kappelhof M Groot AE Lecouffe NE Coutinho JM Yoo AJ. Combined effect of age and baseline Alberta stroke program early computed tomography score on post-thrombectomy clinical outcomes in the MR CLEAN registry. Stroke. (2020) 51:3742–5. doi: 10.1161/STROKEAHA.120.031773 33092478

[7] Jahan R Saver JL Schwamm LH Fonarow GC Liang L Matsouaka RA. Association between time to treatment with endovascular reperfusion therapy and outcomes in patients with acute ischemic stroke treated in clinical practice. JAMA. (2019) 322:252–63. doi: 10.1001/JAMA.2019.8286 31310296 PMC6635908

[8] Beuker C Köppe J Feld J Meyer CL Dröge P Ruhnke T. Association of age with 1-year outcome in patients with acute ischaemic stroke treated with thrombectomy: real-world analysis in 18 506 patients. J Neurol Neurosurg Psychiatry. (2023) 94:631–7. doi: 10.1136/JNNP-2022-330506, PMID: 37001983 PMC10359560

[9] Fahey M Crayton E Wolfe C Douiri A. Clinical prediction models for mortality and functional outcome following ischemic stroke: a systematic review and meta-analysis. PLoS One. (2018) 13:e0185402. doi: 10.1371/journal.pone.0185402, PMID: 29377923 PMC5788336

[10] Ovbiagele B Reeves MJ Nasiri M Johnston SC Bath PM Saposnik G. A simple risk index and thrombolytic treatment response in acute ischemic stroke. JAMA Neurol. (2014) 71:848–54. doi: 10.1001/JAMANEUROL.2014.689, PMID: 24798141

[11] Saposnik G Guzik AK Reeves M Ovbiagele B Johnston SC. Stroke prognostication using age and NIH stroke scale: SPAN-100. Neurology. (2013) 80:21–8. doi: 10.1212/WNL.0b013e31827b1ace, PMID: 23175723 PMC3589202

[12] Almekhlafi MA Davalos A Bonafe A Chapot R Gralla J Pereira VM. Impact of age and baseline NIHSS scores on clinical outcomes in the mechanical thrombectomy using solitaire FR in acute ischemic stroke study. AJNR Am J Neuroradiol. (2014) 35:1337–40. doi: 10.3174/ajnr.A3855, PMID: 24557701 PMC7966577

[13] Ospel JM Brown S Kappelhof M van Zwam W Jovin T Roy D. Comparing the prognostic impact of age and baseline National Institutes of Health stroke scale in acute stroke due to large vessel occlusion. Stroke. (2021) 52:2839–45. doi: 10.1161/STROKEAHA.120.032364, PMID: 34233465

[14] Li Q Abdalkader M Siegler JE Yaghi S Sarraj A Campbell BCV. Mechanical Thrombectomy for large ischemic stroke: a systematic review and Meta-analysis. Neurology. (2023) 101:E922–32. doi: 10.1212/WNL.0000000000207536, PMID: 37277200 PMC10501098

[15] Abderrakib A. Torcida N. Sadeghi N. Naeije G. (2023). Crossed cerebellar diaschisis worsens the clinical presentation in acute large vessel occlusion. Cerebrovasc Dis. 52: 552–559. 36716718 10.1159/000528676

[16] DeLong ER DeLong DM Clarke-Pearson DL. Comparing the areas under two or more correlated receiver operating characteristic curves: a nonparametric approach. Biometrics. (1988) 44:837. doi: 10.2307/2531595, PMID: 3203132

[17] Campbell BCV Majoie CBLM Albers GW Menon BK Yassi N Sharma G. Penumbral imaging and functional outcome in patients with anterior circulation ischaemic stroke treated with endovascular thrombectomy versus medical therapy: a meta-analysis of individual patient-level data. Lancet Neurol. (2019) 18:46–55. doi: 10.1016/S1474-4422(18)30314-4, PMID: 30413385

[18] Goyal M Menon BK van Zwam WH Dippel DWJ Mitchell PJ Demchuk AM. Endovascular thrombectomy after large-vessel ischaemic stroke: a meta-analysis of individual patient data from five randomised trials. Lancet (London, Engl). (2016) 387:1723–31. doi: 10.1016/S0140-6736(16)00163-X 26898852

